# Involved site radiation therapy in stage I-III nasopharyngeal carcinoma with limited lymph node burden (ISRT-NPC) or elective region irradiation: a study protocol for a multicenter non-inferiority randomized controlled phase III clinical trial

**DOI:** 10.1186/s12885-023-11212-7

**Published:** 2023-08-03

**Authors:** Yang Liu, Yaqian Han, Feng Liu, Desheng Hu, Zhijian Chen, Peiguo Wang, Jingao Li, Jiyong Qin, Feng Jin, Yexiong Li, Jingbo Wang, Junlin Yi

**Affiliations:** 1 Department of Radiation Oncology, National Clinical Research Center for Cancer/Cancer Hospital, National Cancer Center, Chinese Academy of Medical Sciences and Peking Union Medical College, 17 Panjiayuan Nanli, Chaoyang District, Beijing, 100021 China; 2 Department of Radiation Oncology, Hubei Cancer Hospital, Wuhan, 430079 Hubei Province China; 3 Department of Radiation Oncology, Hunan Cancer Hospital, Changsha, 410013 Hunan Province China; 4 Department of Radiation Oncology, Guizhou Cancer Hospital, Guiyang, 550000 Guizhou Province China; 5 Department of Radiation Oncology, Jiangxi Cancer Hospital, Nanchang, 330029 Jiangxi Province China; 6 Department of Radiation Oncology, Tianjin Medical University Cancer Institute & Hospital, Tianjin, 300060 China; 7 Department of Radiation Oncology, Yunnan Cancer Hospital, Kunming, 650100 Yunnan Province China; 8 Department of Radiation Oncology, Cancer hospital Chinese academy of medical science, Shenzhen center, Shenzhen, 518127 Guangzhou Province China; 9 Department of Radiation Oncology, National Cancer Center/National Clinical Research Center for Cancer/Hebei Cancer Hospital, Chinese Academy of Medical Sciences (CAMS), Tongxi Road, Guangyang District, Langfang, 065001 Hebei Province China

**Keywords:** Nasopharyngeal carcinoma, Limited lymph node burden, Involved site radiation therapy, Clinical target volume, Regional control, Toxicity, Quality of life

## Abstract

**Background:**

Current radiotherapy guidelines and consensus statements uniformly recommend elective region irradiation (ERI) as the standard strategy for nasopharyngeal carcinoma (NPC). However, given the scarcity of skip-metastasis, the improved assessment accuracy of nodal involvement, and the striking advancements in chemotherapy for NPC, a one-fits-all delineation scheme for clinical target volumes of the nodal region (CTVn) may not be appropriate anymore, and modifications of the CTVn delineation strategy may be warranted. Involved site irradiation (ISI) covering merely the initially involved nodal site and potential extranodal extension has been confirmed to be as effective as ERI with decreased radiation-related toxicities in some malignancies, but has not yet been investigated in NPC. This study aims to compare the regional control, survival outcomes, radiation-related toxicities, and quality of life (QoL) of ISI with conventional ERI in NPC patients with a limited nodal burden.

**Methods:**

ISRT-NPC is a prospective, multicenter, open-label, noninferiority, phase III randomized controlled trial. A total of 414 patients will be randomly assigned in a 1:1 ratio to receive ISI or ERI. Randomization will be stratified by institution scale and N stage. Generally, in the ISI group, the high-risk CTV1 (dose: 60 Gy) includes a 1-cm expansion of the positive LN as well as the VIIa and the retrostyloid space above the bilateral transverse process of the atlantoaxial spine (C1), regardless of N status. The low-risk CTV2 (dose: 50 Gy) covers the cervical nodal region with a 3-cm caudal expansion below the transverse process of C1 for N0 disease and a 3-cm expansion below the positive LN for positive LNs.

**Discussion:**

The results of this trial are expected to confirm that ISI is a non-inferior strategy to ERI in stage I-III patients with low LN burden, enabling the minimization of treatment-related toxicity and improvement of long-term QoL without compromising regional control.

**Trial registration:**

ClinicalTrails.gov, NCT05145660. Registered December 6, 2021.

## Background

Nasopharyngeal carcinoma (NPC) is one of the most common head and neck malignancies. Southeast Asia is the top epidemic region for NPC, with an age-standardized incidence rate of five (per 100,000 population) [1]. Given the complicated anatomical location and high radiosensitivity of NPCs, radiotherapy is the mainstay of treatment for these tumors. The National Comprehensive Cancer Network (NCCN) guidelines recommend radiotherapy alone for stage I NPC and concurrent chemoradiotherapy (CCRT) with or without induction chemotherapy as the standard treatment for stage II-IVA NPC [2].

Intensity-modulated radiotherapy (IMRT) is well-accepted as the preferred radiotherapy technique in treating NPC. In addition, advancements in chemotherapy, such as the regimen optimization of induction chemotherapy and consolidation metronomic chemotherapy, have been extensively investigated and validated to improve the outcome of NPCs, achieving 5-year overall survival (OS) and local-regional control (LC) rates exceeding 90% for stage II-III NPC [3]. These extraordinary survival rates have yielded a population that will experience not only long-term survival but also marked treatment-related sequelae [4, 5]. Therefore, de-escalation of the therapeutic strategy is required for considerably curable malignancies, with the aim of reducing treatment-related toxicity while maintaining excellent cure rates.

Current guidelines recommend at least 70 Gy of radiotherapy to the gross tumor volume (GTV), namely the primary lesion at the nasopharynx as well as involved lymph nodes (LNs) [2]. The clinical target volumes for the nodal region (CTVn) that have been outlined to cover potential sub-clinically involved areas are generally divided into high- (CTVn1, 60–70 Gy), intermediate- (CTVn2, 60 Gy) and low-risk regions (CTVn3, 50–54 Gy) [6]. The international guidelines uniformly recommend that the CTVn2 includes a higher risk of subclinical lesions in all patients with NPC [6]. Nonetheless, these recommendations are not risk-adjusted for nodal location and burden. The results from our institutional real-world study of 2025 patients revealed that the 5-year regional-recurrence-free survival (RRFS) significantly worsened with an increasing nodal burden (97.6%, 97.5%, 94.6% and 91.8% for N0-3 stages, respectively), which confirmed the significant heterogeneity in stage II-IVa patients [7]. Therefore, subjecting everyone to the same treatment modality may be an arbitrary approach. Furthermore, numerous previous studies have reported that most regional recurrences are observed within the nodal GTV or CTVn1 region with high doses and that the marginal or out-of-field failure rates were below 10% [8, 9]. Therefore, lower-intensity cervical irradiation may be justified for early- to mid-stage patients with a relatively low burden of LNs (LB-LN).

As a part of IMRT, elective region irradiation (ERI), defined as prophylactic irradiation of the entire region of involvement plus at least one subsequent level, has become the standard strategy since the two-dimensional (2D) era [10]. However, ERI inevitably leads to acute and late toxicities closely related to quality of life (QoL), including persistent xerostomia, subcutaneous fibrosis, and mouth-opening difficulties [4, 5, 11, 12]. Furthermore, the radiation-related adverse effects on the lymphatic system reduce lymphocyte counts, impair the preservation of immune function, and even promote tumor growth by limiting the adaptive immune response [13]. Additionally, a combination of radiotherapy and chemotherapy exacerbates treatment-related toxicities, which remains an unaddressed point of concern in the IMRT era [3].

In comparison with ERI, involved site irradiation (ISI), a modified modality performed using cervical irradiation covering merely the initially involved nodal site and possible extranodal extension (ENE), has been confirmed to be quite effective in hematologic malignancies with low radiation-related toxicities [14, 15]. Nevertheless, to the best of our knowledge, no study has reported the validity of ISI in NPC. Given the scarcity of skip node metastases in NPC and the increased sensitivity of diagnostic imaging modalities in detecting occult involved LNs, the introduction of ISI for NPC treatment can be considered to be justifiable [16, 17].

Consequently, this study will apply the ISI strategy in early to mid-stage NPC and compare its regional control rates, survival outcomes, radiation-related toxicities, and QoL with the conventional ERI approach. We hypothesize that ISI with appropriate volume reduction of CTVn can reduce treatment-related toxicities and better preserve immune function without compromising regional control.

## Methods

### Study design

**Fig. 1:**
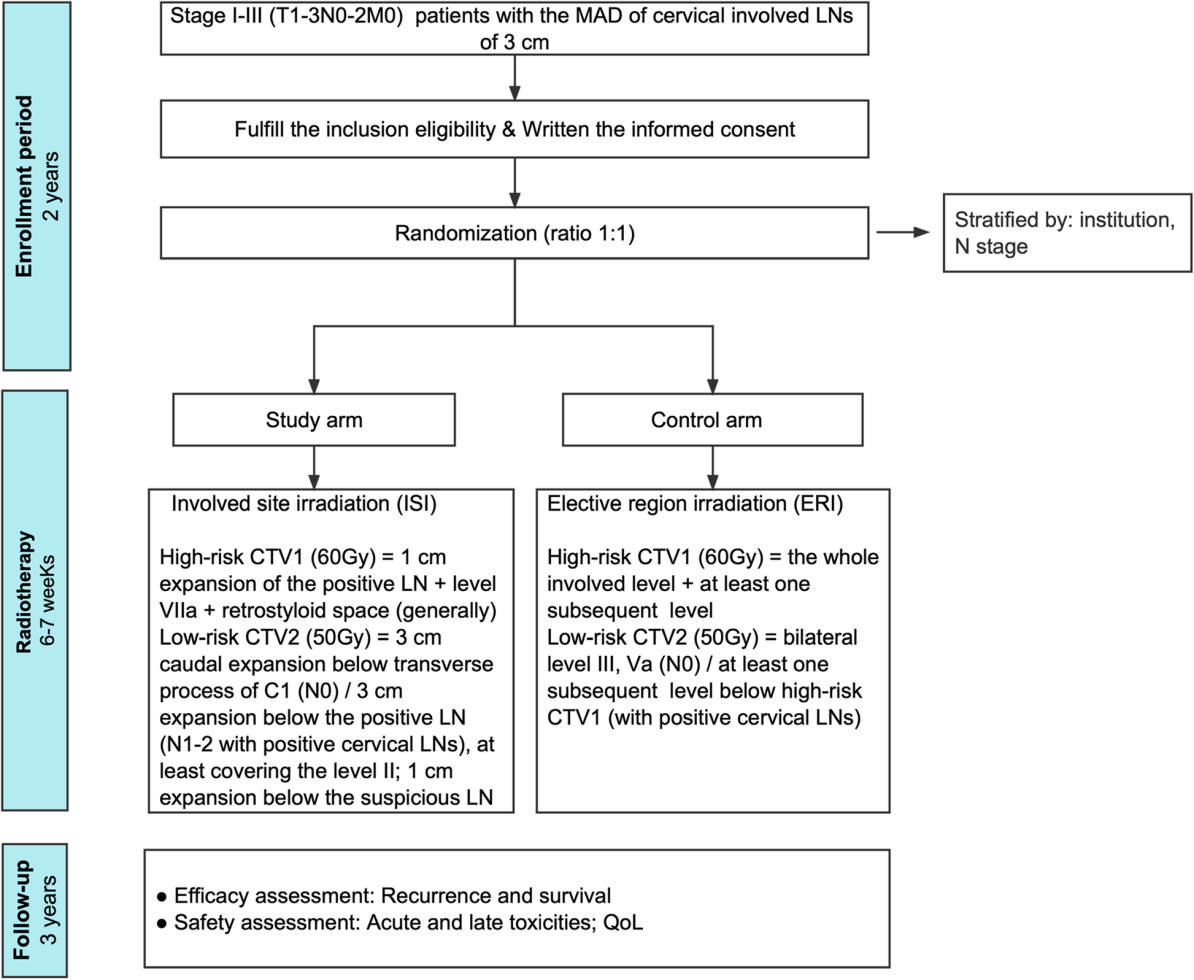
Flow chart showing the overview of this trial. CTV1, high-risk clinical target volume for the nodal region; CTV2, low-risk clinical target volume for the nodal region; C1, the atlantoaxial spine; ENE, extranodal extension; GTV, Gross tumor volume; Gy, gray; LN, lymph node; MAD, maximum diameter; QoL, quality of life; RPLN, retropharyngeal lymph node

This study is designed as a multicenter, noninferiority, subject-blinded, randomized, phase III trial. A total of 414 patients harboring stage I-III NPC with LB-LN (detailed definition is presented in “Inclusion criteria” section) from eight tertiary hospitals in China will be randomized (1:1) to the ISI or ERI groups. The flow chart and study design schedule are presented in Fig. 1; Table 1, respectively.

**Table 1:** Schedule of study assessment

Period	Pre-RT	During RT			within 2 years after RT	3–5 years after RT	5 years after RT
Time		Weekly	Mid	End	every 3 months	every 6 months	annually
Eligibility	X						
Written informed consent	X						
History	X						
Baseline documentation^$^	X						
Physical examination	X	X	X	X	X	X	X
General laboratory tests^#^	X	X	X	X	X	X	X
EBV-DNA measurement	X	X	X	X	X	X	X
Radiological image examinations							
Nasopharyngoscopy	X		X	X	X	X	X
MRI of nasopharynx and neck	X		X	X	X	X	X
Cervical ultrasound	opt		opt	opt	opt	opt	opt
CT of chest and abdomen	X				every 6 months	X	X
Abdominal CT/ultrasound	X				every 6 months	X	X
ECT bone scan	X				annually	annually	X
PET/CT	opt				opt	opt	opt
Toxicities/Adverse events reporting	If related to study procedures	X	X	X	X	X	X
Quality of life	X		X	X	X	X	X

*RT *Radiation therapy, *EBV *Epstein–Barr virus, *CT *Computed tomography, *ECT *Emission computed tomography, *MRI *Magnetic resonance imaging, *PET *Positron emission tomography.

General laboratory tests include blood routine analysis, biochemistry analysis, urine and stool routine analysis, circular lymphocyte phenotyping, and EBV-DNA measurement. $ includes electrocardiography, pulmonary function measurements, and assessments of hypotension and diabetes.

### Primary endpoint

The 3-year RRFS (Regional-recurrence-free survival, which is measured from the registration to the documented regional recurrence) will be compared between the ISI and ERI groups.

### Secondary endpoint

The effects of ISI on 3-year OS (measured from registration to documented death from any cause or last follow-up), distant metastasis-free survival (DMFS, measured from registration to documented distant metastasis or death from any cause), and progression-free survival (PFS, measured from registration to documented locoregional recurrence or distant metastasis or death from any cause); early (from the start of radiotherapy until the 1 month after radiotherapy) and late toxicities; and general and late toxicity-related QoL will be evaluated.

### Inclusion criteria

1. Age between 18 and 75 years;
2. Karnofsky performance status (KPS) score ≥ 70;
3. Pathologically confirmed World Health Organization (WHO) type II-III NPC;
4. TNM stage I-III (T1-3N0-2M0) according to the 8th American Joint Committee on Cancer / Union for International Cancer Control (AJCC/UICC) staging system with a maximum diameter (MAD) of cervical involved LNs ≤ 3 cm [18] and without high-grade ENE [19, 20], namely LB-LN;
5. Available baseline nasopharynx and neck computed tomography (CT) or magnetic resonance imaging (MRI) (strongly advocated) data (including functional MRI sequences) and measurable tumor lesions;
6. All procedures for defining the tumor burden completed within 4 weeks of registration;
7. Survival expectancy of at least 6 months;
8. Normal marrow and organ function: hemoglobin ≥ 120 g/L, WBCs ≥ 4 × 10^9^ /L, platelets ≥ 100 × 10^9^ /L; liver and kidney function-related indicators within 1.5*the normal upper limit;
9. Patient willingness to comply with the protocol;
10. Patient willingness and ability to provide an informed consent form.

### Exclusion criteria

1. MAD of cervical metastatic LNs > 3 cm [18];
2. High-grade ENE of cervical LNs (including matted nodes and LNs infiltrating the adjacent muscle, parotid gland, vessels or skin) [19, 20];
3. AJCC T4 or N3 stage;
4. History of other malignancies (except for stage I non-melanotic skin cancer or in-situ cervical cancer);
5. Pregnant or lactating women or women of childbearing age without contraception;
6. Concurrent enrollment in another interventional clinical trial;
7. Uncontrolled comorbidities that may reduce compliance with the trial, such as myocardial infarction, arrhythmia, cerebrovascular disease, ulcer disease, psychiatric disease, and uncontrollable diabetes;
8. Unwillingness to comply with procedures and requirements as per the study protocol regularly.

### Randomization

An eligibility checklist and patient consent must be completed and obtained before randomization. Eligible patients will be allocated randomly to the ISI and ERI groups on a 1:1 basis using a computer-generated randomization scheme with a block size of four. Randomization will be stratified by institution scale (large vs small) and N stage (N0 vs N1 vs N2).

### Pre-treatment evaluation (baseline)

The enrolled patients are required to complete the following examinations within 4 weeks before registration:

1. Thorough medical history and physical examination: evaluation of KPS score, weight, height, vital signs, and physical examination of the nasopharynx, cervical LNs, and nervous system.
2. Laboratory tests: blood routine examinations, biochemistry evaluations, urine and stool routine analysis, circular lymphocyte phenotyping, and measurement of Epstein–Barr virus (EBV) DNA concentration.
3. Radiological imaging examinations: fiberoptic nasopharyngoscopy and tumor biopsy, MRI and CT contrast imaging of the nasopharynx and neck, cervical ultrasound, chest CT, abdominal CT/ultrasound, emission computed tomography (ECT) bone scans, and positron emission tomography (PET)/CT (optimal; performed at the discretion of the attending physician).
4. Baseline documentation: electrocardiogram, pulmonary function, and QoL assessment.

### Radiotherapy

Simulation: To ensure consistency in the multicenter setting, the localization and immobilization procedures on CT and MRI will be based on the Chinese Consensus Guidelines for Radiotherapy in NPC (version 2020) [21]. In brief, CT and MRI scans will be performed in the supine position with a thermoplastic mask at the head, neck, and shoulder. The scans will be captured in 3-mm slices from the head to 3 cm below the sternoclavicular joint.

**Fig. 2:**
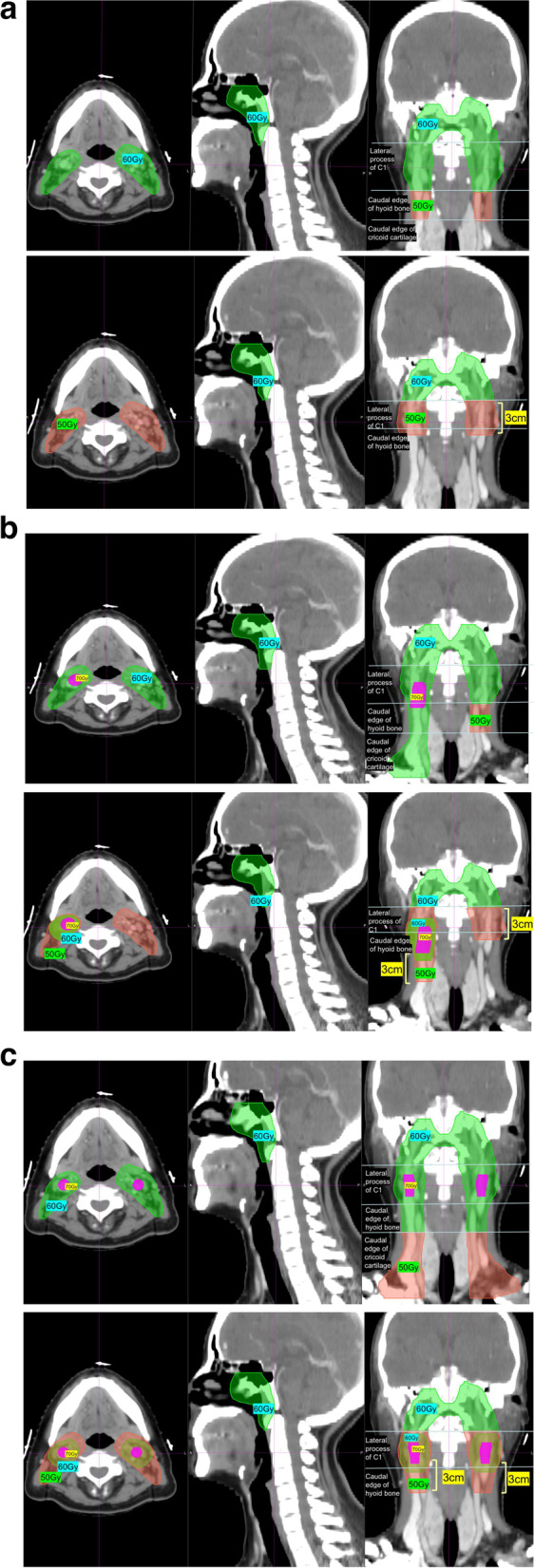
**A** Comparison of CTV delineation between the ISI and ERI groups in patients with N0 disease (Upper panel: ERI; Lower panel: ISI). CTV, clinical target volume; ERI, elective region irradiation; ISI, involved site irradiation. **B** Comparison of CTV delineation between the ISI and ERI groups in patients with ipsilateral positive LNs in level II extending to level III (Upper panel: ERI; Lower panel: ISI). CTV, clinical target volume; ERI, elective region irradiation; ISI, involved site irradiation; LN, lymph node. **C** Comparison of CTV delineation between the ISI and ERI groups in patients with bilateral positive LNs (Upper panel: ERI; Lower panel: ISI). CTV, clinical target volume; ERI, elective region irradiation; ISI, involved site irradiation; LN, lymph node

1. Target volume delineation: The same principles will be shared between the two arms regarding the delineation of GTVnx, GTVnd, and CTV for the primary tumor and organs at risk (OAR). Detailed definition of GTVnd is in accordance with the international guidelines [6]. Suspicious LN is defined as the one with MAD between 5mm and 10 mm as well as ambiguous radiological features. With regard to the CTV for LNs, patients will be randomized to follow the respective strategies of ISI and ERI groups (Table 2). Figure 2 A-C shows the delineation examples of ISI and ERI.

**Table 2:** Delineation of the ISI and ERI groups

	ISI	ERI
GTVnx	Apparent macroscopic primary lesion at the nasopharynx based on detailed physical examination, fiberoptic nasopharyngoscopy, and diagnostic CT, MRI or PET/CT images	
GTVrpn	Positive RPLN	
GTVnd	Positive cervical LN	
High-risk CTV1	GTVnx plus a 10-mm margin to encompass the microscopic extension of the gross tumor, the whole nasopharynx, the high-risk structures recommended for CTVp2 by the contouring consensus* (such as parapharyngeal space, skull base, pterygopalatine fossa and the inferior part of the nasal cavity and maxillary sinus), the VIIa, and the retrostyloid space above the bilateral transverse process of C1.	
N0	Suspicious LN (if available)	Bilateral level II
N1-2	Geometrically 1-cm expansion of the positive LN (GTVnd) or GTVrpn, without exceeding anatomical barriers; Suspicious LN (if available)	The whole involved level + at least one subsequent level
Low-risk CTV2		
N0	Bilateral cervical nodal region with the 3-cm caudal expansion below transverse process of C1 and at least covering the level II; 1-cm expansion of the suspicious LN (if available)	Bilateral level III + Va
N1-2	Ipilateral cervical nodal region with the 3-cm expansion below the positive LN (GTVnd) and at least covering the level II; contralateral cervical nodal region with the 3-cm caudal expansion below the transverse process of C1 and at least covering the level II; 1-cm expansion of the suspicious LN (if available)	At least one subsequent level below CTV1
*PGTVnx, PGTVrpn, PGTVnd, PGTVns, PTV1, PTV2 are generated by geometric 3-5mm expansion of the GTVnx, GTVrpn, GTVnd, GTVns, CTV1 and CTV2, respectively. Abbreviations: CTV *Clinical target volume, * GTV *Gross tumor volume, *LN *Lymph node, *ISI *Involved site irradiation, *ERI *Elective region irradiation, *nx *nasopharynx, *rpn/RPLN *retropharyngeal lymph node, *ns* suspicious LN

3. Dose prescription and suggested dose constraints of OARs: As per the latest ASCO/CSCO and NCCN guidelines, dose prescription is shown in Table 3. Dose constraints of OARs are based on those recommended in the 2020 international guideline on dose prioritization for NPC [22].

**Table 3:** Dose prescription

Target volume	Total dose (Gy)	Fraction dose (Gy)	Fractions
PGTVnx	69.96	2.12	33
PGTVrpn	69.96	2.12	33
PGTVnd	69.96	2.12	33
PGTVns	60.06	1.82	33
PTV1	60.06	1.82	33
PTV2	50.96	1.82	28
*PGTV *Planning gross tumor volume, *GTV *Gross tumor volume, *PTV *Planning target volume, *nx* nasopharynx, *rpn *retropharyngeal lymph node			

### Chemotherapy

#### Concurrent chemotherapy

For stage III patients, concurrent cisplatin (100 mg/m², d1-3, Q3w, maximum to three cycles) will be administrated with radiotherapy. For stage II patients, the use of concurrent chemotherapy will be determined by the discretion of physicians. T2N0-1 patients with all nodes < 3 cm, no level IV or Vb nodes involvement, no extranodal extension [ENE] and pretreatment Epstein-Barr virus [EBV] DNA < 500 copies/mL may be considered as candidates of RT alone. Stage I patients will receive radiotherapy alone.

#### Induction and consolidation chemotherapy

The application of induction chemotherapy and consolidation chemotherapy is dependent on the physician’s discretion.

#### Follow-up

During the treatment course, all enrolled patients will be examined and treatment-related adverse events will be carefully documented weekly. Patients will be followed up at least every three months in the first two years, six months in the third to fifth year, and then yearly. The detailed schedule and examination items are shown in Table 1.

#### Assessment of recurrence

In patients showing signs of local recurrence during follow-up, MRI, CT, PET-CT or ultrasound-guided fine-needle aspiration cytology (US-FNAC) should be performed to confirm recurrence, as necessary. The planning CT and diagnostic imaging modalities will be compared side-by-side to determine the exact site of recurrence and its relative location to the ISI region.

#### Assessment of toxicity and QoL

Acute treatment-related toxicities will be graded using the National Cancer Institute Common Terminology Criteria for Adverse Events (CTCAE) version 5.0. The Radiation Therapy Oncology Group (RTOG) and European Organization for Research and Treatment of Cancer (EORTC) late radiation morbidity scoring schemes will be used to assess late radiation toxic effects. The EORTC QLQ-C30 and EORTC QLQ-H&N35 questionnaires will be applied to evaluate the general QoL. The Groningen Radiation Therapy Induced Xerostomia questionnaire (GRIX) will be used to assess the QoL associated with serostomia. The MD Anderson Dysphagia Inventory (MDADI) composite score and Swallowing Quality of Life Questionnaire (SWAL-QoL) will be used to evaluate swallowing capacity and dysphagia-related QoL.

#### Sample size calculation

The primary endpoint is RRFS. Based on previous reports, the 3-year failure free regional control rate was assumed to be 96% in both groups [23]. According to expert consensus, data from institutional experiences, and published literature, a 6% difference was set as the noninferiority margin [3, 7, 24]. To obtain a power of 85% and a one-sided α value of 2.5%, a total of 414 patients would be enrolled with a dropout or loss of follow-up rate of 5%.

### Statistical analysis

The case distribution between the ISI and ERI groups in each center will be described. Compliance will be assessed according to the case report form to compare implementation between the two groups. Total drop-out rates and adverse event drop-out rates will be compared between the two groups using Fisher’s exact test. Baseline comparisons will be performed by using *t*-tests or non-parametric tests for continuous data. Chi-square test will be used to compare categorical data. Both intention-to-treat (ITT) and per-protocol (PP) analysis will be used to assess the efficacy of ISI. Safety analysis will be performed in the safety population. Time-to-event data will be censored in the absence of observations of regional failure at the date of last follow-up or loss to follow-up. The Kaplan–Meier method will be used to estimate the survival rates. Differences between treatment groups will be assessed using log-rank tests. Interaction analysis for RRFS will be undertaken to assess whether differential effects were present between ISI and ERI in predefined subgroups. Adverse events will be listed and analyzed using the chi-square test or Fisher’s exact test. Serious adverse events should be listed in detail and compared between groups. The principal investigator and the protocol committee will perform the analysis. A Data Monitoring Committee (DMC) will be set up to oversee the trial and to decide whether the trial should be stopped.

### Ethics

The study protocol has been approved by the ethics committee of the Cancer Hospital, Chinese Academy of Medical Sciences (22/107-3308). The study has been registered in ClinicalTrails.gov (NCT05145660).

### Trial status

The Recruitment started in August 2022 and is currently ongoing.

## Discussion

The target volume delineation consensus for NPC still recommends uniform CTVn borders across the different N categories [6, 25]. Moreover, the current recommendations are based on the anatomic landmarks easily identifiable during surgery, such as the hyoid bone and cricoid cartilage [26]. However, the actual lymphatic drainage should have not been restricted by the anatomical structures; theoretically, it is more likely to be affected by the size of the LN as well as the distance from the foci of the tumor deposit. Therefore, the subclinical target of the nodal region in NPC remains a matter of debate for the radiation oncologists.

Extensive studies on CTVn volume reduction have mostly focused on omitting elective contralateral or lower-neck irradiation and have confirmed the safety, feasibility, and improved long-term QoL of this approach [24, 27–29]. Chen et al. conducted a prospective study of 212 patients with clinical N0-1 NPC and demonstrated the safety and efficacy of omitting level IV and Vb [27]. The study by Tang et al. consisting of 546 NPC patients with unilateral neck LN metastases reiterated the feasibility of contralateral lower-neck-sparing IMRT [30]. Similar results were reported in a study by Gao et al., who limited the nodal clinical target volume (CTV) to level II, III, and Va in 410 patients with cN0 NPC [31]. Notwithstanding these lower-neck sparing efforts, substantial late toxicities were still reported, such as persistent xerostomia, subcutaneous fibrosis, open mouth difficulty, decreased QoL, and radiation-related damage to the lymphatic system [4, 5, 11, 12, 32]. A recent randomized phase III noninferiority study demonstrated that elective upper-neck irradiation (UNI) of the uninvolved neck provided similar regional control and results in less radiation toxicity than standard whole-neck irradiation (WNI) in patients with N0-N1 NPC [24]. Nevertheless, the suitability of the above approach for patients with ipsilateral N2-3 disease and non-endemic populations requires further investigation. Moreover, these studies still depended heavily on the anatomical definition of the neck region without considering the specific site of LN. Therefore, much effort is required to further optimize the nodal CTV delineation strategy in NPC.

ISI, which is characterized by coverage of only the initially involved nodal sites and high-risk region, has been shown to reduce treatment-related toxicity without compromising high regional control rates in various hematological malignancies [14, 15]. This approach has not been tested in NPC due to concerns regarding the possibility of missing suspected metastatic LN. Nevertheless, ISI may be a qualified alternative to ERI in NPC based on the following considerations. First, skip involvement of cervical nodes in NPC is scarce [16]. Second, the majority of regional failures in cases of NPC mainly occur in high-dose areas within the irradiation field, suggesting that failure may be attributable to primary resistance to radiotherapy rather than inadequate target volume coverage [8, 9]. Third, the increased sensitivity of imaging modalities has made clinically inappreciable small nodal metastases detectable and resulted in a very low incidence of occult nodal metastases [17]. All of the abovementioned conditions allow for the hypothesis of further reduction of the volume of neck irradiation in low-risk NPC.

The most accurate delineation of the CTV is based on true extension of subclinical disease. However, complete pathologic examination of the lymphoid adipose tissue is impossible in NPC. Therefore, to determine the potential spread distance of LNs alongside the neck, we will investigate the potential drainage distance by measuring the distance of one positive lymph node surrounded by another positive lymph node. By marking the centers of the two most caudally located LNs in 73 patients with more than two involved cervical LNs, two coordinates ([X_1_, Y_1_, Z_1_] and [X_2_, Y_2_, Z_2_]) were obtained. The |Z_1_-Z_2_| value (caudal-cranial direction) was calculated, and the value of 2.96 cm was set as the potential caudal expansion distance by covering 95% of the patients (unpublished data). Additionally, according to the CTV delineation consensus for N + patients with head and neck cancer, CTV3 (elective dose CTV) was recommended to include nodal areas at least 2 cm cranial and caudal to GTV-N [33]. On the basis of these considerations, 3 cm below the GTVnd was set as the extended border for CTV2 [33].

Currently, there is no consensus regarding the microscopic extent of the disease or the CTV margin that should surround the involved gross node, i.e., determination of the best margin to find between CTVn1 and CTVn2 away from the long-established practice remains unknown. This can be attributed to the paucity of surgical pathology data regarding the exact area of extracapsular tumor infiltration. On the basis of common recommendations in current guidelines and considering the critical findings that no microscopic tumor extension beyond 10 mm has been observed among LNs involved in head and neck cancers (none-NPC), coupled with the common practice of the major centers in this trial, we will expand the GTVn by 10 mm to cover the CTVn1 [34]. In addition, since ENE is widely reported to be a more powerful determinant of poor survival outcomes, patients with high grade of ENE will not be included in this study [35]. Although no significant differences were observed between nodal size and ENE extension distance in head and neck cancers, MAD plays a critical prognostic role in NPC, and an MAD of 3 cm is widely used as an essential negative determinant of worse RRFS [18]. Herein, we will carefully evaluate the size and ENE status of nodes to determine enrollment and will exclude patients with MAD > 3 cm or high-grade ENE to balance potential confounding factors.

In conclusion, the results of this trial are expected to confirm that ISI is a safe and effective strategy to reduce neck irradiation volume in comparison with ERI in stage I-III patients with low LN loads, minimizing treatment-related toxicity and improving long-term QoL without compromising regional control.

## Data Availability

The datasets used and analysed during the current study will be presented within the manuscript and the additional supporting files.

## Abbreviations

C1: Atlantoaxial spine
CCRT: Concurrent chemoradiotherapy
CTCAE: Common Terminology Criteria for Adverse Events
CTV: Clinical target volume
DMFS: Distant metastasis-free survival
EBV: Epstein-Barr virus
ECT: Emission computed tomography
ENE: Extranodal extension
EORTC: European Organization for Research and Treatment of Cancer
ERI: Elective region irradiation
GRIX: Groningen Radiation Therapy Induced Xerostomia questionnaire
GTV: Gross tumor volume
IMRT: Intensity-modulated radiotherapy
ISI: Involved site irradiation
ITT: Intention-to-treat
KPS: Karnofsky performance status
LC: Local-regional control
LN: Lymph nodes
MAD: Maximum diameter
MDADI: MD Anderson Dysphagia Inventory
NCCN: National Comprehensive Cancer Network
NPC: Nasopharyngeal carcinoma
OAR: Organs at risk
OS: Overall survival
PET: Positron emission tomography
PFS: Progression-free survival
PP: Per-protocol
PTV: Planning target volume
QoL: Quality of life
RPLN: Retropharyngeal lymph node
RRFS: Regional-recurrence-free survival
RTOG: Radiation Therapy Oncology Group
UNI: Upper-neck irradiation
WHO: World Health Organization
WNI: Whole-neck irradiation
